# Supplementary material for: Chromothripsis during telomere crisis is independent of NHEJ, and consistent with a replicative origin
Source: Genome Res. 2019 May;29(5):737–49. doi: 10.1101/gr.240705.118 (PMC6499312; doi:10.1101/gr.240705.118)
Supplement: Supplemental Material [file supp_gr.240705.118_Supplemental_file_1.zip › contigs/annotated_contigs/DB113/contig.6.DB113_length_1227_mean_cov_27.4474327628.docx]

**DB113_length_1227_mean_cov_27.4474327628**

```
CCTTGCTTCAAATGTGGTTGTGCCGCTTGCTGGCTACGTAGCTGTTTTCCTTTTTAAGGCAGAGATACTGATAATGAGAAAAAGCATGG
 >chr10:12952247-12952599 + E=1e-199
TGTATTTGGTGTGCCTTAAAGCCATTCAATGTCGAATGCAGTTGCTGCTACATATTGCCAGACTCAGTCCATAACCATATGTCCCCAAA

TAAATTCTCAGTTAATATTTTATTTGGTAAAAATATTATTGGAAAACCTATAAACAACAGATGACAAGCACAATGATACGTGTTTCTGC

CAATTCAAGGATTGTCCTTCCTGGCTTGGTATCTGGCACAAGTCCTTGCAAACTTGCAAGTTTCAATAACTAGTCAGAGCAAAAA|CAT
                                                                                       >
GTGTTTTTTGGCTGCATAAATGTCTTCTTTTGAGAAGTGTCTGTTCATATCCTTTGCCCACTTTTTGATGGGGTTGTTTGCTTTTTTCT
chr10:13120133-13120253 - E=4e-60 p=2e-03
TATAAATTTGTTTAAGTTCTTTGTAGAT|A|CTCTTTTTTGGTTCCATATGAACTTTAAAGTAGTTTTTTCCAATTCTGTGAAGAAAGT
                              >chr2:133211537-133211596 + E=2e-23
A|GAAATAACTGAAAAACTCAT|GTTTTTACTGCCATAGCCCTAGTGCCTAATACTGTGC|TGGAGTATTGTC|ACAGGGCAAGACAGT
                      >chr10:12953303-12953340 + E=3e-10               >chr10:1231604
CTCAAAAAAAAAAGAAAGAAAAGAAAAAGAAAGCTTGAGAAAAATGGGATCTTTTAGATACGATTCAGAGGTACTCACAATAAGCGTAA
4-12316248 + E=2e-100
CAGTTAGTACCAAGAAGTGACTGCTCCTGGCCAGCTGAGCTGCAGCCAAGGACAGCAAACAAGGAAGGGACCTGATAGCCCAGGCAAAC

TGTTTTAA|TCCTTG|CTGGATTTCAAAATTGCTATGGACCAGTAACTTCTGTGTACCACCCATTTTCAACCTTTTTGAGTAGGAATGT
               >chr10:13065575-13065994 - E=7e-234
CTATTGCAGGTTTCCTATGCTTGCCTTATCGTTGTATATTGGGTGTGTGTAGGCCAGAAAACACACCTCTTTAGTTCACACATCTTCAG

ATAAAGAGGCGTTGCACCCAAGAGACCCCTTCTGCACCTGGATTGATTTGGATGATGAGATTCTGTACATGATATCATAATGAGACAAG

ACCTCGGATGCTTTGGAGGGGATAATTATATTTTTCATGTAGTAAGGACATGGTCCATTAGAGACCAGAGAGTGCTCTGGTGGCCCTGA

ATGAATCTTATTTCCTGGGATTCAGAGCTTTGTGTAGTTCCCTCCATCATTGACTCTCAGCTTGGCCACGTGACTAGCT
```
